# Safety of flow-controlled ventilation with positive and negative end-expiratory pressure in a swine model of intracranial hypertension

**DOI:** 10.1186/s40635-024-00703-x

**Published:** 2024-12-13

**Authors:** Álmos Schranc, John Daniels, Roberta Südy, Fabienne Fontao, Philippe Bijlenga, Guillaume Plourde, Hervé Quintard

**Affiliations:** 1 Unit for Anaesthesiological Investigation, Department of Anaesthesiology, Pharmacology, Intensive Care and Emergency Medicine, University of Geneva, 1 rue Michel-Servet, Geneva, Switzerland; 2 Division of Neurosurgery, Department of Clinical Neurosciences, Geneva University Hospitals, 4 rue Gabrielle-Perret-Gentil, Geneva, Switzerland; 3 Division of Intensive Care Medicine, Department of Medicine, Centre Hospitalier de l’Université de Montréal, 1051 rue Sanguinet, Montréal, Canada; 4 Division of Intensive Care, Department of Anaesthesiology, Pharmacology, Intensive Care and Emergency Medicine, Geneva University Hospitals, 4 rue Gabrielle-Perret-Gentil, Geneva, Switzerland

**Keywords:** Flow-controlled ventilation, Positive end-expiratory pressure, Negative end-expiratory pressure, Cerebral hemodynamics, Intracranial hypertension, Mechanical ventilation

## Abstract

**Background:**

Patients with brain damage often require mechanical ventilation. Although lung-protective ventilation is recommended, the application of increased positive end-expiratory pressure (PEEP) has been associated with elevated intracranial pressure (ICP) due to altered cerebral venous return. This study investigates the effects of flow-controlled ventilation (FCV) using negative end-expiratory pressures (NEEP), on cerebral hemodynamics in a swine model of intracranial hypertension.

**Methods:**

A model of intracranial hypertension involving bilateral trepan bolt holes was performed in 14 pigs. Pressure-controlled volume-guaranteed ventilation (PCV-VG) with PEEP and FCV using PEEP and then NEEP were applied. Intracranial pressure and oxygenation, as well as systemic hemodynamics and gas exchange parameters, were continuously monitored. Data were collected at baseline and at varying PEEP levels for both PCV-VG and FCV ventilation modalities. Following this, FCV ventilation and NEEP levels of -3, -6 and -9 cmH_2_O were applied.

**Results:**

ICP remained stable with low PEEP levels, but significantly decreased with NEEP. Lower ICP following NEEP improved cerebral perfusion pressure and cerebral tissue oxygenation (*p* < 0.05 for all). FCV with NEEP at EEP-6 and EEP-9 significantly improved cardiac output and mean arterial pressure (MAP), compared to PCV-VG and FCV using PEEP (*p* < 0.05, respectively). There were no significant differences in gas exchange parameters between modalities (PCV-VG vs FCV), and between the application of PEEP or NEEP. No significant correlations were observed between ΔICP and ΔMAP.

**Conclusion:**

The application of FCV with NEEP appears to be a safe ventilation mode and offers an additional tool for controlling severe intracranial pressure episodes. These findings warrant validation in future studies and may lead to important potential applications in clinical practice.

**Supplementary Information:**

The online version contains supplementary material available at 10.1186/s40635-024-00703-x.

## Introduction

Many patients with brain trauma require mechanical ventilation when admitted to the intensive care unit (ICU) to ensure adequate oxygenation and CO_2_ clearance [1, 2]. Recently published guidelines concerning patients with acute brain injury (ABI) propose the application of lung-protective ventilation settings to minimize the effect of ventilation on intracranial pressure (ICP) [3, 4]. Nonetheless, the optimal setting for positive end-expiratory pressure (PEEP) has yet to be determined. In a recent trial on ABI patients, the elevation of PEEP from 5 to 15 cmH_2_O resulted in a significant increase in ICP [5], probably due to decreased cerebral venous return induced by increased intrathoracic pressure [6]. Thus, applying high PEEP to comply with protective ventilation guidelines without further increasing ICP is a challenging conundrum for clinicians caring for ABI patients [4, 5, 7].

Flow-controlled ventilation (FCV) using the Evone® ventilator (Ventinova Medical, Eindhoven, Netherlands) is a relatively new ventilation modality involving a constant inspiratory and expiratory flow [8]. As a consequence of the active expiratory phase, which uses the Venturi effect [9], the ventilator can be set to apply negative end-expiratory pressures (NEEP). Although this setting could possibly lead to lung de-recruitment and deterioration of gas exchange, it may also result in improved venous return and decreased intrathoracic pressure. This could in turn lead to a reduction in ICP. Thus, FCV with NEEP may represent an alternative ventilation strategy for ABI patients during elevated ICP episodes.

Hence, we aimed at characterizing the effects of FCV at various NEEP levels (−3, −6, and −9 cmH_2_O) on cerebral and systemic hemodynamic parameters in a swine model of intracranial hypertension (ICH) and comparing them to a conventional ventilation modality (pressure-controlled ventilation-volume guaranteed (PCV-VG)). We also aimed at comparing the effects of FCV to PCV-VG using varying PEEP levels.

## Methods

### Ethics

The experimental protocol was approved by the Animal Welfare Committee of the Canton of Geneva and the Experimental Committee of the University of Geneva, Switzerland (No. 34446/GE172A-B, 6 March 2023). All procedures were performed in accordance with current Swiss animal protection laws (LPA, RS455). The current report follows the Animal Research Reporting of In Vivo Experiments (ARRIVE) guidelines [10].

### Preparation of animals

Fourteen large-white pigs (male = 7, female = 7, 51.9 ± 4.4 kg) were purchased from the farm supplying the University (Markus Stirnimann, Apples, VD, Switzerland), and were delivered at least two days before the experiments to allow for adaptation to the new environment. The pigs were fasted 12 h before the experiments but had access to water ad libitum. The experiments were held between January and July 2023.

### Mechanical ventilation and monitoring

Following the premedication of the animals with an intramuscular injection of Haloperidol (0.15 mg/kg), Midazolam (0.75 mg/kg) and Atropine (12.5 µg/kg) in the animal pen, the animals were transported to the operating room breathing spontaneously. The oxygen saturation of the animals during transportation to the experimental room was > 96%. A peripheral venous line was secured allowing for the injection of fluids and drugs. The animals were then pre-oxygenated with FiO_2_ 1.0 through a veterinary facial mask with spontaneous breathing for a period of 3 min and then anesthesia induction was performed with an i.v. injection of Fentanyl (2.5 µg/kg) and the inhalation of Sevorane (up to 3%). During this period (another 3 min) the animals were hand ventilated. Following anesthesia induction and iv. administration of rocuronium (1 mg/kg), the animals were intubated and ventilated using PCV-VG with standard ventilatory settings (tidal volume [Vt]: 7 mL/kg; respiratory rate [RR]: 30–35/min; inspired fraction of oxygen [FiO_2_]: 0.4; inspiratory-to-expiratory time ratio [I:E ratio]: 1:2) on an EVITA XL INFINITY ventilator (Draeger, Lubeck, Germany). Anesthesia and analgesia were ensured by continuous infusions of propofol (10–15 mg/kg/h), fentanyl (2–4 mcg/kg/h) and rocuronium (0.5–1 mg/kg/h). During both PCV-VG and FCV ventilation, the level of applied end-expiratory pressure varied from + 3 to + 9 cmH_2_O. FCV ventilation was performed with standard ventilatory settings (flow: 12–15 L/min to achieve the same respiratory rate as PCV-VG and maintain normocapnia; peak pressure was set to achieve a Vt of 7 mL/kg; FiO_2_: 0.4; I:E ratio: 1:1). FCV ventilation also included a phase with applied NEEP at levels of −3, −6 and −9 cmH_2_O. A conventional tube adaptor supplied by the manufacturer allowed for changes in ventilation modalities without manipulating the endotracheal tube (ETT).

The femoral artery and vein were cannulated using the conventional Seldinger technique under ultrasound guidance. The arterial and venous partial pressure of oxygen (PaO_2_, PvO_2_) and carbon-dioxide (PaCO_2_, PvCO_2_) were determined from simultaneously collected arterial and central venous blood samples (VetScan i-STAT1, Abaxis, Union city, CA, USA). Tracheal pressure, heart rate and the electrocardiogram were recorded by specific sensors (PowerLab, ADinstruments, Oxfordshire, UK). Mean arterial pressure (MAP), heart rate (HR) and cardiac output (CO) were measured continuously (PiCCO, Pulsion Medical Systems, Munich, Germany) with a calibration via transpulmonary thermodilution occurring at every change in ventilation modality. Body temperature was measured with a rectal thermometer (Thermalert TH-8, Physitemp, Clifton, NJ, USA) and maintained at 38 ± 0.5 °C using a heating pad (Mio Star, Zurich, Switzerland). To avoid bias in the measurements, catheters were not placed in the neck veins to preserve a normal outflow from the brain unhindered by large bore catheters placed in a jugular vein.

Intracranial pressure, cerebral temperature and cerebral regional tissue oxygenation (PtiO_2_) were measured continuously using a specific probe (Raumedic AG, Helmbrechts, Germany) inserted 20 mm deep into the cortex through a dedicated bolt hole and secured with the manufacturers holding clamp.

### Intracranial hypertension model

We used a previously described intracranial hypertension model, in which two trepan bolt holes are drilled bilaterally 20 mm anterior to the coronal suture and 15 mm lateral from the sagittal suture [11]. One of the bolt holes is used for ICP, temperature and PtiO_2_ measurements, while the other is used for inserting an 8 Fr. 2-way pediatric latex Foley catheter (Well Lead Medical, China). The proximal end of the Foley catheter is positioned approximately 5 mm under the internal table of the skull. When inflated, the distal balloon increases intracranial pressure proportional to the degree of inflation. This setting enables a gradual controlled increase in intracranial pressure that can be stabilized at the desired ICP level. Cerebral perfusion pressure (CPP) was calculated as MAP-ICP [12].

### Study protocol

After insertion of the cerebral catheters and a 60-min stabilization period, ICP and PtiO_2_ were measured. The Foley catheter balloon was then inflated with 2–5 mL of NaCl 0.9% solution until the ICP reached > 20 mmHg but < 25 mmHg. The catheter was then attached to a pressure bag equipped with an infusion clamp to allow a constant infusion flow (0.1 ml/h) for maintenance of the intracranial pressure level with minimal manipulation of the system. Data collection began after a one-hour stabilization period of ICP readings.

**Fig. 1:**
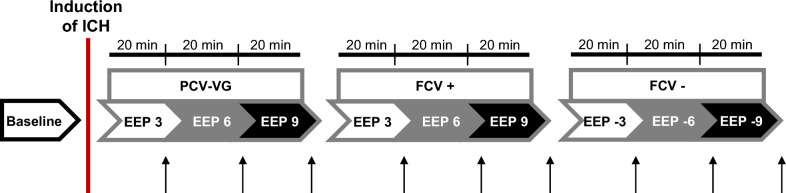
Schematic representation of the experimental protocol. Abbreviations: pressure-controlled volume-guaranteed ventilation (PCV-VG); flow-controlled ventilation with positive end-expiratory pressures (FCV +); flow-controlled ventilation with negative end-expiratory pressures (FCV−); intracranial hypertension (ICH); end-expiratory pressure (EEP, which positive in case of PCV-VG and FCV+ ; and negative in case of FCV−). The arrows indicate measurement points

The animals were ventilated with a PCV-VG mode or a FCV mode for 20-min-long steps at increasing PEEP levels (+ 3, + 6, + 9 cmH_2_O). Finally, the animals were ventilated with an FCV mode, using decreasing NEEP levels (−3, −6, −9 cmH_2_O). Data collection occurred at baseline and at the end of each end-expiratory pressure step. Although hemodynamic and cerebral parameters were monitored continuously, only the mean values of the last 30 s were registered and analyzed. In order to standardize the volume history, a recruitment maneuver (peak pressure of 20 cm H_2_O for 8–10 s) was applied at the beginning of the protocol and following changes to the ventilatory mode. To prevent potential lung derecruitment subsequent to ventilator change, the ETT was clamped on disconnection and the change time to the alternative ventilator kept at less than 8 s. As mentioned above, after every change of modality, a held inspiratory pause allowed for the standardization of the volume history. At the end of the protocol, the animals were euthanized by a single intravenous injection of sodium pentobarbital (100 mg/kg). The scheme of the protocol is summarized in Fig. 1.

### Statistical analyses

Data are expressed as box-plots and mean ± standard deviation (SD). The Shapiro–Wilk test was used to test normality. One-way repeated-measures ANOVA with Holm–Šidák post hoc analysis was used to explore the effects of ventilation modalities and EEP levels. One-way ANOVA with Holm–Šidák post hoc tests were applied to determine the differences in hemodynamics between the ventilation modalities. Statistical analyses were conducted with a significance level of *p* < 0.05. All statistical tests were performed with GraphPad Prism (Version 9.2.0). Considering an alpha risk of 0.05 and a power of 80%, we calculated that 14 pigs would be required to detect a clinically significant 20% decrease in ICP from baseline (prospective calculation for a repeated measures ANOVA, G*Power3). All the experimental animals were included in the final data analysis.

## Results

### Impact of FCV on the cerebral hemodynamic profile

**Fig. 2:**
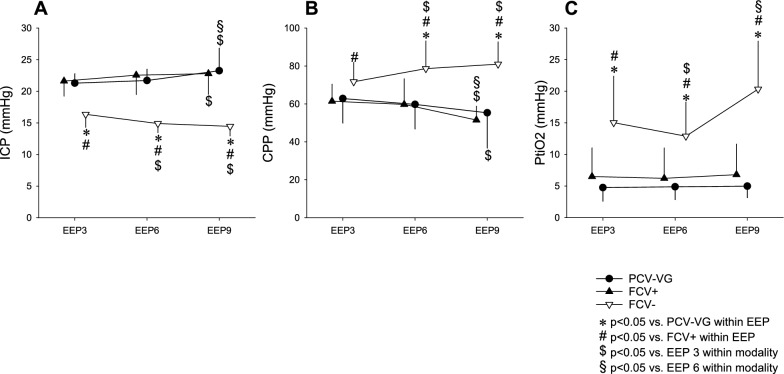
Cerebral hemodynamic parameters such as intracranial pressure (ICP - panel A), cerebral perfusion pressure (CPP - panel B), cerebral regional tissue oxygenation (PtiO2 panel - C) during different ventilation modalities and end-expiratory pressures. Abbreviations: pressure-controlled volume-guaranteed ventilation (PCV-VG); flow-controlled ventilation with positive end-expiratory pressures (FCV +); flow-controlled ventilation with negative end-expiratory pressures (FCV−), end-expiratory pressure (EEP, which positive in case of PCV-VG and FCV + ; and negative in case of FCV−). *:* p* < 0.05 vs. PCV-VG within EEP; #:* p* < 0.05 vs. FCV + within EEP; $:* p* < 0.05 vs. EEP3 within modality, §:* p* < 0.05 vs. EEP6 within modality

The effect of different ventilation settings on cerebral hemodynamics is summarized in Fig. 2. At baseline, ICP was 21 ± 1.4 mmHg, CPP was 64 ± 16 mmHg and PtiO_2_ was 10.0 ± 8.9 mmHg. We observed no variations in ICP related to increases in PEEP from + 3 to + 6 cmH_2_O either with PCV-VG or FCV (*p* > 0.05 for all; Fig. 2, Graph A). At a PEEP of + 9 cmH_2_O, ICP increased in both modalities (*p* < 0.05, respectively; Fig. 2, Graph A). A significant decrease in ICP was observed when FCV with NEEP was applied from −3 to −9 cmH_2_O (22 ± 1.5 vs. 15 ± 1.5 mmHg; *p* < 0.001; Fig. 2, Graph A). This decrease in ICP was associated with a significant increase in the CPP (54 ± 8 vs. 80 ± 13 mmHg; *p* < 0.001; Fig. 2, Graph B). PtiO_2_ improved significantly when FCV with NEEP was applied from −3 to −9 cmH_2_O (15.0 ± 7.3 vs. 20.3 ± 7.6 mmHg; *p* = 0.0025), whereas no variation in PtiO_2_ was observed between any ventilation strategy using PEEP (*p* > 0.05 for all; Fig. 2, Graph C).

### Impact of FCV on gas exchange and systemic hemodynamics

**Fig. 3:**
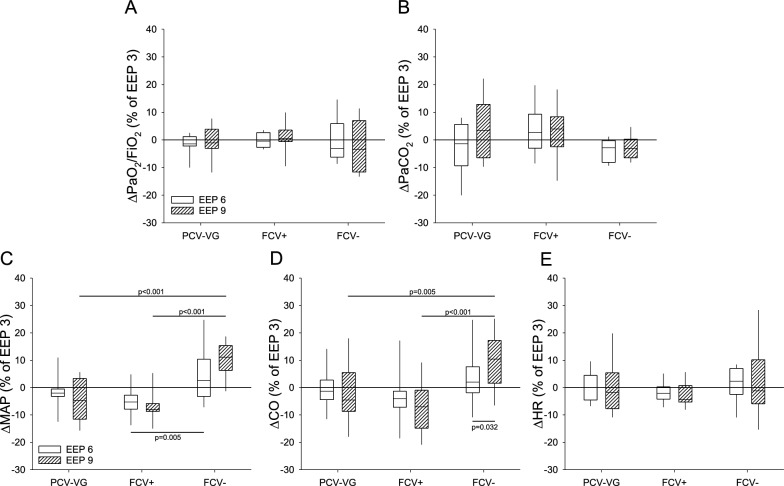
Relative changes to EEP3 in oxygenation index (PaO_2_/FiO_2_ - panel A), arterial partial pressure of carbon-dioxide (PaCO_2_ - panel B), mean arterial pressure (MAP - panel C), cardiac output (CO - panel D) and heart rate (HR - panel E). The parameters were observed under the following ventilation modalities: pressure-controlled volume-guaranteed ventilation (PCV-VG); flow-controlled ventilation with positive end-expiratory pressures (FCV +); flow-controlled ventilation with negative end-expiratory pressures (FCV−)

Gas exchange and systemic hemodynamic parameters observed during PCV-VG and FCV at baseline and PEEP or NEEP levels of 3 cmH_2_O are presented in Table 1. The relative changes related to the different end-expiratory pressure levels are summarized in Fig. 3.

**Table 1:** Gas exchange and hemodynamic parameters (mean ± SD) such as oxygenation index (PaO_2_/FiO_2_), arterial partial pressure of carbon-dioxide (PaCO_2_), mean arterial pressure (MAP), cardiac output (CO) and heart rate (HR) under baseline (BL) conditions and at 3 cmH_2_O end-expiratory pressure (EEP 3) during pressure-controlled volume-guaranteed ventilation (PCV-VG), flow-controlled ventilation with positive EEPs (FCV +) and flow-controlled ventilation (FCV−)

		PaO_2_/FiO_2_ (mmHg)	PaCO_2_ (mmHg)	MAP (mmHg)	CO (L/min)	HR (1/min)
BL		480 ± 43	40 ± 5	94 ± 17	5.4 ± 0.7	99 ± 8
EEP 3	PCV-VG	491 ± 55	38 ± 7	83 ± 12^†^	5.4 ± 0.7	92 ± 10
	FCV +	484 ± 51	41 ± 6	84 ± 12^†^	5.2 ± 0.8	90 ± 8^†^
	FCV−	459 ± 67	43 ± 8	88 ± 9	5.4 ± 1.1	84 ± 10^†^

^†^: *p* < 0.05 vs. BL

At baseline and during the experimental protocol PaO_2_/FiO_2_ and PaCO_2_ were in the normal range (Table 1 and Fig. 3, Graph A and B), and ventilatory modes with PEEP as well as NEEP showed no significant effect on any gas exchange parameters (*p* > 0.05 for all).

Improved MAP and CO values were observed during FCV with NEEP-6 and −9 cmH_2_O, compared to those in PCV-VG and FCV using PEEP (*p* < 0.05, respectively; Fig. 3, Graphs C and D). HR was independent of ventilation modality or PEEP/NEEP level when elevated ICP was present (Fig. 3, Graph E).

## Discussion

In a model of pigs with intracranial hypertension, we report that FCV appears to be a safe alternative when mechanical ventilation is required. We observed that “low” PEEP (< 10 cmH_2_O) had no impact on ICP. We also observed, for the first time, that the use of NEEP via FCV mode was associated with a significant decrease in ICP, with a simultaneous improvement in CPP and PtiO_2_. Systemic hemodynamic parameters such as MAP and CO were also improved using NEEP, without any deleterious effects on systemic oxygenation parameters.

The use of PEEP has been challenged recently in the ABI population [3, 4]. With the application of PEEP, jugular vein outflow is reduced, venous return is decreased, and cardiac output and blood pressure are reduced. All of these have a detrimental impact on the CPP, independently of the intracranial pressure level [13]. A recent clinical paper reported that a PEEP level over 10 cmH_2_O, could affect a significant change in ICP and CPP [14]. In our study, we observed a significant effect of PEEP on ICP only at 9 cmH_2_O.

NEEP improved CO and MAP in our animal model. Although we did not measure the total intravascular volume, we can expect that this improvement was due to an increased venous return since heart rate did not change. We also observed that NEEP significantly decreased ICP at all tested NEEP levels. This phenomenon could be explained in different ways:

Firstly, there may be a facilitating effect on venous return through decreased intrathoracic pressure that is confirmed by the increase in stroke volume and cardiac output observed at the same time. Secondly, autoregulation may play a role. As previously described, the PEEP level has no effect on the autoregulation mechanism [5, 15, 16]. Similarly, during NEEP, there appears to be minimal effect on the autoregulation mechanism (Figure S1). Indeed, MAP variations were not correlated, whatever the level of end-expiratory pressure (EEP), with variations in ICP. We could expect that the lowered ICP values observed with the application of NEEP could be the result of the combined effect of increased venous return with increased MAP, and activation of the autoregulation vasoconstriction response [17–19]. In these conditions, increases in MAP and cardiac output occurring with the application of NEEP, could decrease intracranial pressure through the autoregulation response.

We reported a significant improvement of cerebral tissue oxygenation after NEEP. As observed by Giardina et al., we report no impact of the PEEP level on PaO_2_ [5]. In our model, using healthy animals, we observed no variation of PaO_2_ with any NEEP levels. Thus, improvement of PtiO_2_ was not linked to the improvement of respiratory parameters of lung aeration, but rather in relation to the observed increase in hemodynamic parameters, such as MAP or CO. However, we have to underline the fact that PtiO_2_ was low at baseline (< 20 mmHg) even after careful calibration. These low levels of PtiO_2_ have already been described in pig models [11, 20, 21]. In these conditions, interpretation of PtiO_2_ improvement after FCV using NEEP must be cautious, limited to observing the trend values.

This study has several limitations. Firstly, we decided to use animals with no lung injury. In clinical practice, nearly 20% of brain-injured patients present respiratory failure occurring during hospitalization [4]. Respiratory failure is common during the acute phase, secondary to lung aspiration or ventilator acquired pneumonia. In these conditions, compliance and respiratory parameters could be altered and the effects of PEEP or NEEP could potentially be very different. Robba et al. described that using higher values of PEEP could be safe and not necessarily increase ICP further if lung injury warrants its use and PEEP did not promote hypotension [3]. It would be of interest to further explore the effects of NEEP applied to a mixed model of brain injury associated with respiratory failure.

Secondly, we reported an absence of any deleterious effect on gas exchange parameters, using FCV and NEEP. However, the ventilatory time in the NEEP mode was short (20 min for each level of NEEP, 60 min for total NEEP ventilation), and thus cannot predict any potential deleterious effects that could become present with prolonged mechanical ventilation in this model. It would likewise be interesting to further investigate oxygenation and lung aeration during prolonged ventilation with this ventilatory mode (FCV + NEEP), in a mixed model of brain and lung injury.

The impact of the different ventilation modes and end-expiratory pressures on cerebral autoregulation was only assumed indirectly (see online data supplement), determining the correlation between the changes of mean arterial pressure and intracranial pressure (changes from 3 cmH_2_O to 9 cmH_2_O and changes from −3 cmH_2_O to −9 cmH_2_O). Cerebral flow was not directly measured, and the pressure reactivity index was not calculated. Both of these may be more direct markers of cerebral autoregulation.

Another limitation of this study is that, in accordance with the 3R Principles, which aims at reducing the number of animals used in experimentation, a self-control design was used in this investigation. As such, we were unable to perform any histological analysis that would be specific to an individual mode of ventilation. Furthermore, the different ventilation modalities and end-expiratory pressures might possibly have affected the results of the next ventilation session. This effect could have been reduced with a randomization and cross-over design, however, this additional switch between respirators would have increased the risk of atelectasis development. Therefore, we used a standard order of ventilation, and the carryover effect was minimized by the implementation of hyperinflation maneuvers (inspiratory pause over 10 s), before every ventilation phase to standardize volume history.

Finally, we were unable to compare FCV-NEEP to another ventilator allowing for a NEEP setting through an ETT tube. This is because no other ventilator is commercially available that allows for a NEEP mode, due to the fact that NEEP can only be applied if the ventilator has an active expiratory phase, as does the Evone device. This is an additional limitation to our study.

## Conclusion

We report, for the first time, that FCV with NEEP up to −9 cmH_2_O allowed for a rapid and significant decrease in ICP when compared to a conventional ventilatory mode, in a swine model of increased intracranial pressure. The use of FCV and the NEEP function may present a rescue therapy for controlling life-threatening episodes of severe increased intracranial pressure. These findings warrant validation in future studies such as investigating the long-term effects of NEEP on gas exchange, respiratory mechanics and lung parenchyma. In addition, the use of NEEP as a method to control elevated ICP levels needs to be studied in the context of a mixed model of elevated ICP and lung injury, as neurotrauma patients often present with some form of lung injury during their ICU stay. Furthermore, the beneficial hemodynamic effects of NEEP should be explored further in both clinical and translational settings, particularly in cases of hemodynamic instability. The findings from such studies would provide valuable insights into the safe application of this modality in critically ill intensive care patients.

## Supplementary Information

**Additional file 1.**

## Data Availability

The datasets used and/or analyzed during the current study are available from the corresponding author on reasonable request.

## Declarations

### Presentation

Preliminary data of this study were presented as a poster at the Annual Congress of the European Respiratory Society in 2023.
