# Supplementary material for: Safety of flow-controlled ventilation with positive and negative end-expiratory pressure in a swine model of intracranial hypertension
Source: Intensive Care Med Exp. 2024 Dec 13;12:117. doi: 10.1186/s40635-024-00703-x (PMC11645394; doi:10.1186/s40635-024-00703-x)
Supplement: Supplementary file 1 — Additional file 1. [file 40635_2024_703_MOESM1_ESM.docx]

**Safety of flow-controlled ventilation with positive and negative end‑expiratory pressure in a swine model of intracranial hypertension**

***Online data supplement***

Álmos Schranc^1^, John Daniels^1^, Roberta Südy^1^, Fabienne Fontao^1^, Philippe Bijlenga^2^, Guillaume Plourde^3^, Hervé Quintard^4^

^1^ Unit for Anaesthesiological Investigation, Department of Anaesthesiology, Pharmacology, Intensive Care and Emergency Medicine, University of Geneva, 1 rue Michel-Servet, Geneva, Switzerland

^2^ Division of Neurosurgery, Department of Clinical Neurosciences, Geneva University Hospitals, 4 rue Gabrielle-Perret-Gentil, Geneva, Switzerland

^3^ Division of Intensive Care Medicine, Department of Medicine, Centre Hospitalier de l’Université de Montréal, 1051 rue Sanguinet, Montréal, Canada

^4^ Division of Intensive Care, Department of Anaesthesiology, Pharmacology, Intensive Care and Emergency Medicine, Geneva University Hospitals, 4 rue Gabrielle-Perret-Gentil, Geneva, Switzerland

**Impact of FCV on cerebral autoregulation**

In order to describe the effects of the different modalities on cerebral autoregulation, we realized a non-parametric correlation test (Spearman) between ΔICP and ΔMAP. The ΔICP and ΔMAP values were calculated as the changes in ICP and MAP corresponding to differing EEP levels: -3 cmH_2_O to -9 cmH_2_O and 3 cmH_2_O to 9 cmH_2_O.

No correlation between ∆ICP and ∆MAP was noted when FCV with NEEP was applied at levels of -3 to -9 cmH_2_O (p=0.755; Figure S1, Graph A), and in FCV and PCV-VG when PEEP was changed from +3 to +9 cmH_2_O (p=0.369; Figure S1, Graph B).


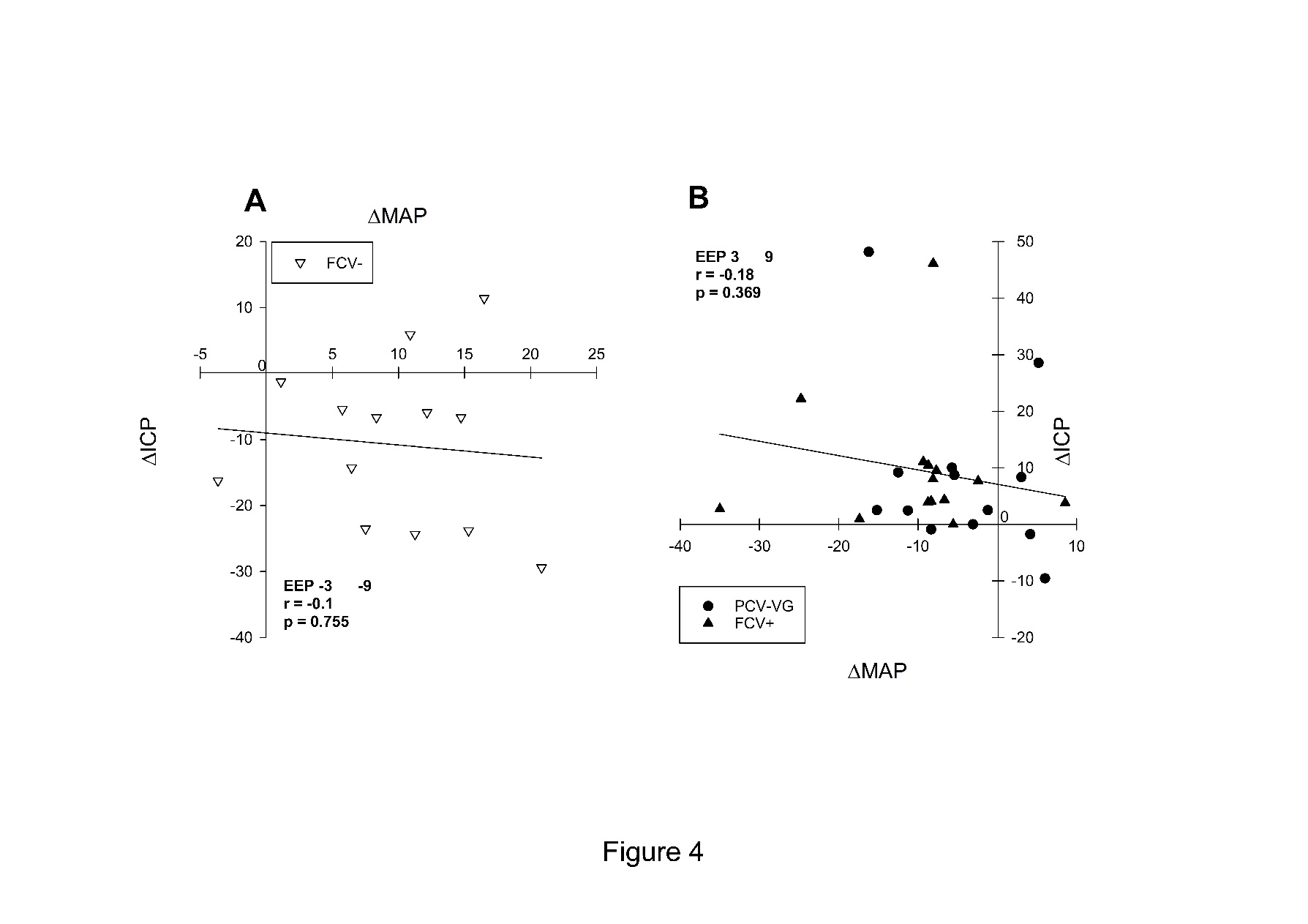


***Figure S1:*** *Correlation between changes in intracranial pressure (∆ICP) and mean arterial pressure (∆MAP). The ΔICP and ΔMAP values were calculated as the changes between EEP levels from -3 cmH_2_O to -9 cmH_2_O and from 3 cmH_2_O to 9 cmH_2_O. These changes were observed under the following ventilation modalities: pressure-controlled volume guaranteed ventilation (PCV-VG); flow-controlled ventilation with positive end-expiratory pressures (FCV +); flow-controlled ventilation with negative end-expiratory pressures (FCV -).*
